# A retrospective clinical study of comparing paclitaxel plus S-1 versus paclitaxel plus cisplatin as the first-line treatment for patients with advanced esophageal squamous cell carcinoma

**DOI:** 10.18632/oncotarget.13602

**Published:** 2016-11-25

**Authors:** Hai-ying Wang, Zhi-hua Yao, Hong Tang, Yan Zhao, Shui-ling Jin, Wen-ping Zhou, Shu-na Yao, Shu-jun Yang, Yan-yan Liu, Su-xia Luo

**Affiliations:** ^1^ Department of Medical Oncology of Henan Cancer Hospital, Zhengzhou University Affiliated Cancer Hospital, Zhengzhou, Henan, China; ^2^ Department of Internal Medicine, The Second Affiliated Hospital of Zhengzhou University, Zhengzhou, Henan, China

**Keywords:** advanced esophageal squamous cell carcinoma, Paclitaxel, S-1, cisplatin, palliative chemotherap

## Abstract

**Background:**

In advanced esophageal squamous cell carcinoma (ESCC), paclitaxel plus cisplatin are considered as active and tolerable. The current clinical study was conducted to retrospectively compare the efficacy and safety of first-line paclitaxel/S-1(PS) and paclitaxel/cisplatin(TP) regimens in advanced ESCC.

**Results:**

The overall response rate of PS was slightly, but not significantly, higher (25 patients, 46%) than that of TP (23 patients, 39%, *P* = 0.432). Median overall survival (OS) was similar for PS and TP (11.5 months vs. 10.4 months, *p* = 0.37). However PS had longer median progression-free survival than TP (PFS: 5.5 months vs5.0months, *p* = 0.04). When compared with PS, more grade 3 or 4 adverse events were recorded for TP, including leukopenia, neutropenia, anemia, anorexia and vomiting (*P* < 0.05). No treatment-related deaths were recorded in either group.

**Patients and Methods:**

Between 2008 and 2014, all patients diagnosed with advanced ESCC and treated with paclitaxel/S-1 or paclitaxel/cisplatin at Cancer Hospital Affiliated to Zhengzhou University were analyzed retrospectively. One hundred and thirteen patients were included in this study. Disease control rates and progression-free survival (PFS) and overall survival (OS) were recorded. Survival analysis was calculated by using Kaplan–Meier method.

**Conclusions:**

The PS option improves PFS and its OS is similar to TP. Moreover, the PS regimen is an effective and safe first-line treatment for ESCC with less hematological and non-hematological toxicity.

## INTRODUCTION

Esophageal cancer is the eighth most common cancer around the world and about 50% of cases occur in China [1, 2]. Surgery is the only potentially curative treatment for localized cancer. Nevertheless, the majority of patients are diagnosed at the advanced stage and have missed the chance of radical surgery [3].With a median survival time of 7–10 months, the prognosis for such patients remains unsatisfactory [4]. As for recurrent or metastatic esophageal cancer, chemotherapy is still the primary cornerstone [5].Although the most commonly used schedules recommended by National Comprehensive Cancer Network guideline are paclitaxel (PTX) or docetaxel (TXT) plus carboplatin/DDP, or 5-fluorouracil (Fu) plus cisplatin (DDP)/oxaliplatin (OXA), no schedule has demonstrated clinical long-term outcome benefiting over the others [6].Median survival has improved gradually, which however is still less than 1 year. In addition, standard treatment remains a matter of debate.

As an novel oral anticancer drug, S-1 consists of tegafur (a prodrug of 5-FU), 5-chloro-2,4-dihydropyrimidine (called gimeracil) and potassium oxonate in a molar ration of 10:0.4:1 [7]. Gimeracil antagonizes dihydropyrimidine dehydrogenase (DPD) and inhibits 5-FU degeneration. Therefore, high concentrations of FU are maintained in serum and tumors for prolonged periods. Potassium oxonate blocks FU phosphorylation in the digestive tract and decreases digestive tract toxic effects [8].Therefore, orally administered S-1 mimics continuous-infusion of 5-FU produces fewer side effects when compared with conventional 5-FU [9]. Accumulating evidence suggests good efficacy and acceptable tolerability of S-1 in various solid tumors, such as advanced gastric cancer [10], colorectal cancer [11], non-small-cell lung cancer [12], pancreatic cancer [13] and head and neck cancer [14].Based on these studies, S-1 is widely applied in Asia for gastrointestinal cancers treatment [15], which has been recently approved in the EU for advanced gastric cancer treatment in combination with cisplatin [8].

Not only as a single agent [16], but also in combination with cisplatin [17, 18], paclitaxel has been reported to yield a good response to advanced esophageal cancer and achieve a median survival time of more than 12 months. Although a number of clinical trials have demonstrated the efficacy and safety of paclitaxel/S-1 regimen in gastric cancer treatment, its application in the first-line setting for ESCC has not been reported [19–24]. Therefore, a study was designed to evaluate the efficacy and safety of paclitaxel/S-1 versus paclitaxel/cisplatin as the first-line treatment for advanced ESCC.

## RESULTS

### Patient characteristics

A total of 976 consecutive medical records were examined. Those who had esophageal adenocarcinoma were excluded. From April 2008 and May 2014, a total of 113 metastatic ESCC patients were enrolled in this retrospective study. Among them, 54 patients were treated with paclitaxel and S-1 regimen, while 59 patients were treated with paclitaxel and DDP. Demographics and baseline characteristics of these patients are summarized in Table 1. Patient characteristics were similar between the two arms. The mean age was respectively 56 years (range: 30–76 years) and 54 years (range: 38–75 years) in the PS group and the TP group. 93% of patients had an ECOG performance status of 0–1. All patients were evaluated for drug efficacy and toxicity.

**Table 1:** Patient baseline characteristics

Baseline characteristics	PS (*n* =54)		TP (*n* = 59)		*p*-value (χ^2^)
	No. of patients (%)		No. of patients (%)		
Age ,years					0.751
Median	56		54		
Range	30–76		38–75		
< 65	45	83	47	80	
≥ 65	9	17	11	20	
Gender (sex)					0.589
Male	34	63	40	68	
Female	20	37	19	32	
ECOG performance status					0.478
0	38	70	47	80	
1	14	26	11	18	
2	2	4	1	2	
Tumor grade					0.466
Poor differentiated	25	46	28	47	
Moderate differentiated	7	13	10	17	
Well differentiated	21	39	17	29	
Unknown	1	2	4	7	
number of metastatic sites					0.782
1	17	31	22	37	
2	27	50	28	47	
≥3	10	19	9	16	

Abbreviations: PS, S-1-paclitaxel; TP, paclitaxel-DDP; ECOG, Eastern Cooperative Oncology Group.

### Treatment

The overall treatment is summarized in Table 2. The median number of cycles received was respectively 5 and 4 in the PS group (range: 3–8) and the TP group (range: 2–8), which was not significantly different (*P* = 0.735). Dose reduction occurred in 9 PS patients (17%) and 15 TP patients (25%). In addition, treatment delays of more than 7 days occurred in 17 PS patients (31%) and 26 TP patients (44%). A similar proportion of patients received the second-line chemotherapy (PS = 65%; TP = 68%).

**Table 2:** Overall treatment summary

	PS (*n* = 54)	TP(*n* = 59)	*P*-value
Treatment administration			
Median number of cycle (range)	5 (3–8)	4 (2–8)	0.735
Dose reductions, patients (%)	9 (17%)	15 (25%)	0.256
Cycle delays (> 7days), patients (%)	17(31%)	26 (44%)	0.169
second-line chemotherapy	34(65%)	40 (68%)	0.589

Abbreviations: PS, S-1- paclitaxel; TP, paclitaxel-DDP;

### Efficacy

**Figure 1:**
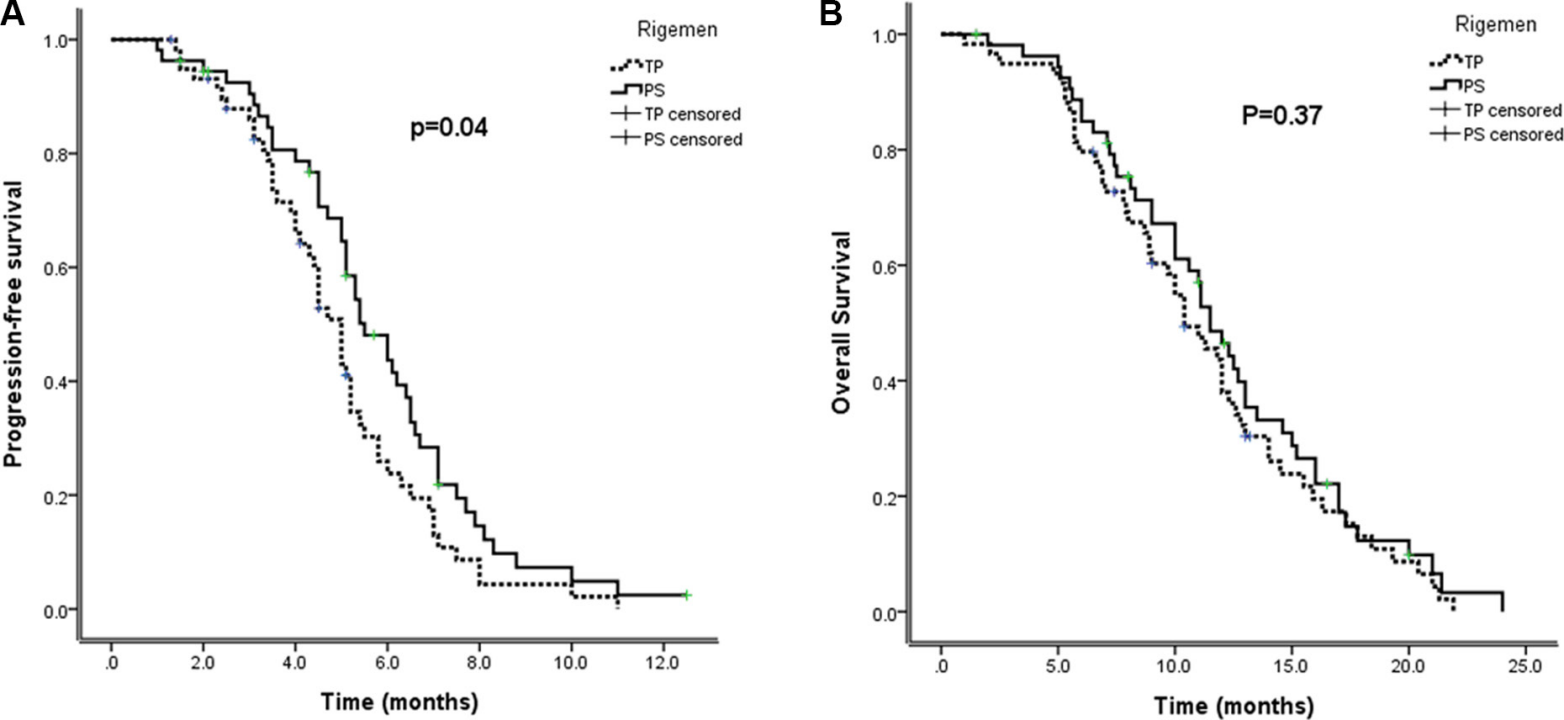
(A) progression-free survival and (B) overall survival by treatment arm (Kaplan–Meier curve) PS,S-1-paclitaxel; TP, paclitaxel-DDP.

The response rate and disease control rate were respectively 46% and 70% (25 PR and 13 SD) in PS and 36% and 66% (2 CR, 21 PR and 16 SD) in TP (Table 3). RR and DCR in the PS group were slightly, but not significantly, higher than that in the TP group (*P* = 0.432). The median follow-up duration was 24 months (range: 3.0–39 months). The median PFS was respectively 5.5 months for PS (95% CI, 4.65–6.35) and 5.0 months for TP (95% CI, 4.54–5.46) groups (*p* = 0.04) (Figure 1A). Besides, the median OS was 11.5 months (95% CI, 10.1–12.9) for the PS group and 10.4 months (95%CI, 8.6–12.2) for the TP group (*p* = 0.37) (Figure 1B, Table 4).

**Table 3:** Overall response to treatment

Tumor Response	paclitaxel + S-1 (*n* = 54)		paclitaxel + DDP (*n* = 59)		*P*-value
	No.	%	No.	%	
Complete response	0	0	2	3	
Partial response	25	46	21	36	
Stable disease	13	24	16	27	
Progressive disease	16	30	20	34	
RR	25	46	23	39	0.432
DCR	38	70	39	66	0.627

RR, response rate; DCR, disease control rate.

**Table 4:** Kaplan–meier analysis

	paclitaxel+ S-1median (95% CI)	paclitaxel+DDPmedian (95% CI)	*p*-value(log rank)
PFS, months	5.5 (4.65–6.35)	5.0 (4.54–5.46)	0.040
OS, months	11.5 (10.1–12.9)	10.4 (8.6–12.2)	0.370

PFS, progression-free survival; OS, overall survival.

### Adverse events

All patients were assessable for toxicity and toxicity profiles of the two regimens are summarized in Table 5. More grade 3 or 4 adverse events were recorded, including neutropenia, febrile neutropenia, anemia, anorexia and vomiting for TP than for PS (*P* < 0.05). When compared with TP, grade 3 or 4 hyperpigmentation was more frequently observed in PS (*P* < 0.0001). Non-hematologic toxicities were generally mild and manageable. In terms of the incidence of thrombocytopenia, febrile neutropenia, peripheral neuropathy and fatigue, there were no remarkable differences between the two treatment groups. Moreover, no unexpected serious adverse reactions or treatment-related deaths were observed in either treatment group. Serious adverse events were more frequently observed in TP than in PS.

**Table 5:** Toxicity profile

Toxicity	Number of patients, *n* (%)						*P*-value (χ^2^)
paclitaxel + S-1 (*n* =54)			paclitaxel+ DDP (*n* = 59)		
Grade	0	I + II	III + IV	0	I + II	III + IV	
Haematological toxicity							
Leukopenia	32 (59)	16(30)	6(11)	25(42)	20(34)	14(24)	0.117
Neutropenia	26 (48)	20(37)	8(15)	13(22)	30(51)	16(27)	0.012
Febrile neutropenia	45(83)	7(13)	2(4)	37(63)	13(22)	9(15)	0.033
Thrombocytopenia	34(63)	14(26)	6(11)	34(58)	18(31)	7(11)	0.837
Anemia	27(50)	20(37)	7(13)	16(27)	31(53)	12(20)	0.043
Non-haematological toxicity							
Anorexia	33(61)	15(28)	6(11)	22(32)	28(51)	9(17)	0.038
Nausea/vomiting	26(48)	28(52)	0	16(27)	38(65)	5(8)	0.013
Diarrhea	40(74)	10(19)	4(7)	51(86)	7(12)	1(2)	0.083
Stomatitis	49(91)	5(9)	0	52(88)	7(12)	0	0.652
Peripheral neuropathy	51(94)	3(6)	0	54(92)	5(8)	0	0.542
hyperpigmentation	25(46)	23(43)	6(11)	51(86)	8(14)	0	< 0.0001
ALT/AST	44(81)	10(19)	0	45(76)	14(24)	0	0.496
Creatinine	51(94)	3(6)	0	54(92)	5(8)	0	0.542
Arthralgia	50(93)	4 (7)	0	56(95)	3(5)	0	0.611
Fatigue	24(44)	28(52)	2(4)	24(41)	32(54)	3(5)	0.645

## DISCUSSION

The incidence of esophageal cancer varies considerably among different populations and geographic regions. Adenocarcinoma is more common in western countries, while squamous cell carcinoma accounts for more than 95% of esophageal cancers in China [25]. Furthermore, NCCN guidelines recommendation concerning esophageal cancer is based on the results of clinical trials that have included some patients with gastroesophageal junction or gastric adenocarcinoma [26, 27].However, ESCC can be differentiated from esophagogastric adenocarcinoma [28].Our study is to find effective regimens based on a homogenous cohort of patients with advanced ESCC.

Preclinical models suggest that the combined use of S-1 and paclitaxel has showed additive to synergistic antitumor effects on gastric cancer and breast cancer *in vitro* [20, 29]. With response rates of 40%–70% and median survival of 11–17 months, a number of clinical trials of S-1 and paclitaxel combination have demonstrated promising results in advanced gastric cancer [19, 21].According to this preclinical and clinical work, a multicenter randomized phase II study showed that in terms of response rate of 50%, the paclitaxel/S-1 arm was non-inferior when compared with paclitaxel/5-FU arm (response rate = 28.3%), disease control rate (68.9% vs 60.6%) and progression-free survival (153 vs 129d, respectively), which compared paclitaxel/S-1 with paclitaxel/5-FU in advanced gastric cancer [23].Reported by E Mochiki et al., another prospective phase II randomized trial showed that in advanced gastric cancer, survival benefit of paclitaxel/S-1 group was non-inferior to that of S-1/cisplatin group with higher RR (52.3% vs 48.7%; *P* = 0.74), longer median PFS (9 vs 6 months; *P* = 0.50) and a OS (16 vs 17 months; *P* = 0.84), but significantly less toxicity [22]. A meta-analysis indicated that when compared with the PTX plus 5-FU therapy, paclitaxel plus S-1 therapy had nearly equivalent safety and a better DCR in advanced gastric cancer [24].As paclitaxel/S-1 has shown significantly improved efficacy in advanced gastric cancer treatment, whether paclitaxel/S-1 can achieve good efficacy in advanced ESCC is wondered.

However, the efficacy of S-1 for metastatic ESCC has been described in very few reports. With an overall response rate of 74.1%, median PFS of 7.7 months and median OS of 16.0 months, concurrent chemoradiotherapy in combination with S-1 plus cisplatin showed high efficacy and good safety for locally advanced or metastatic esophageal squamous cell [30]. The only retrospective study of S-1 as the second or third-line therapy in ESCC patients demonstrated a high tumor response rate of 25%, DCR of 60%, PFS of 100 days (95% CI 75.9–124.1) and median survival of 330 days (95% CI, 278.4–381.6) [31].Moreover, adverse events were mild and acceptable.

To the best of our knowledge, this is the first study of paclitaxel/S-1 doublet for a homogenous cohort of advanced ESCC patients. In the present study, the objective response rate and DCR of the PS group was even slightly higher than those of the TP group (46% versus 36% *P* = 0.432; 70% versus 66% *P* = 0.627). Median overall survival (OS) was similar for PS and TP(11.5 months vs. 10.4 months, *p* = 0.37), however PS had longer median progression-free survival (5.5 months vs. 5.0 months, *p* = 0.04) than TP. In our study, survival outcomes of paclitaxel/S-1 are largely consistent with the results of other platinum-based or taxane-based regimens in advanced ESCC patients [6, 17, 32].These results are similar to those reported in previous studies [33–35].More than one third of patients received second-line chemotherapy. The majority of those were doublet or single agent chemotherapies. Due to poor performance status and short survival, very few patients received three or further lines.

Not only efficacy but also toxicity are important factors when selecting a therapeutic method. In the present trial, good tolerability observed with paclitaxel/S-1 combination is noteworthy [19, 21, 23].Our results reveal that paclitaxel/S-1 combinations are relatively well tolerated. Both haematological and gastrointestinal toxicities are considerably less frequent in PS than in TP arm. On account of the oral formulation of S-1 without intravenous infusion, another advantage is greater convenience. However, cisplatin has several important drawbacks, such as high incidences of nausea, vomiting and renal toxicity negatively affecting patients’ life quality. Adverse effects are often substantial, especially with cisplatin-based regimens.

There are several limitations in this study. Firstly, it was a retrospective review. Secondly, it was performed on a small sample size at a single institution using an initial non-comparative design, which reduced the accuracy of comparisons between the two arms. Thirdly, QoL was not evaluated since the study was retrospective.

In conclusion, our results indicate that as first-line chemotherapies, PS and TP are both effective and feasible for advanced ESCC. However, with relatively favorable safety profiles, PTX in combination with S-1 is a promising and tolerable non-platinum-based regimen. Therefore, chemotherapy regimens without platinum compounds serve as a new alternative for first-line treatment for advanced ESCC. With larger sample sizes, further randomized trials may be useful to assess the role of PS in advanced ESCC.

## MATERIALS AND METHODS

### Patient characteristics

One hundred and thirteen patients who had undergone chemotherapy for advanced ESCC at the Cancer Hospital Affiliated to Zhengzhou University from April, 2008 to May, 2014 were recruited in this study, including 54 patients who received paclitaxel (80 mg/m2) intravenously on days 1, 8 and S-1 orally on days 1–14 within a 21-day cycle chemotherapy in the therapy group. Fifty-nine patients who received paclitaxel (80 mg/m^2^) on day 1, 8 and DDP 75 mg/m^2^ intravenously on day 1 within a 21-day cycle as the control group. Inclusion criteria were histologically confirmed advanced ESCC, over 18 years of age, performance status of 0–2 through the Eastern Cooperative Oncology Group criteria , no previous chemotherapy or radiotherapy and adequate liver, kidney and bone marrow functions. Patients were excluded provided that any of the following conditions were fulfilled: previous chemotherapy; second malignancy; severe ascites requiring drainage; active infection; symptomatic brain metastases and parallel radiation therapy. Before the treatment, a written informed consent regarding chemotherapy drug and toxicity was obtained from all patients.

Clinical variables including age, sex, tumor grade, number of metastatic sites, chemotherapy regimens, median number of cycle,dose reductions,cycle delays and second-line chemotherapy were collected. Blood sample at baseline, before and after each cycle of treatment should be collected for the measurement of white blood cell count,absolute neutrophil count, hematoglobin, and platelet count. We also recorded the changes of transaminase and creatinine levels. The study was approved by the Institute Review Board of the Cancer Hospital Affiliated to Zhengzhou University.

### Safety and outcome assessment

Tumor response was evaluated by computed tomography scans according to Response Evaluation Criteria in Solid Tumor (RECIST) criteria 1.1 [36]. Disease control was defined as complete remission (CR), partial remission (PR), or stable disease (SD). Patients who had a progression disease after two cycles of treatment were defined as progression disease (PD). PFS was defined as the time from the first day of treatment to the date of progressive disease or the date of death from any cause. OS was defined as the time from the first day of treatment to the date of death from any cause. Toxicity was graded according to the United States National Cancer Institute's common toxicity criteria(version 2.0) [37].

### Statistical analysis

All of the statistics analyses were performed using SPSS version 14.0 (SPSS Inc., Chicago, Ill., USA). All of the tests were two-sided, and *P* < 0.05 was considered statistically significant. Descriptive variables of patient characteristics and toxicities were directly calculated from the database. χ^2^ test and Fisher's exact test were adopted to compare toxicities and response in the two groups.

With the use of the Kaplan–Meier method, survival curves were constructed for PFS and OS. Median survival and its 95% confidence interval (CI) were predicted. In addition, the log-rank test was employed to compare PFS and OS between treatment groups.
